# Cannabinoids and Terpenes as an Antibacterial and Antibiofouling Promotor for PES Water Filtration Membranes

**DOI:** 10.3390/molecules25030691

**Published:** 2020-02-06

**Authors:** Ismara Nadir, Nosheen Fatima Rana, Nasir Mahmood Ahmad, Tahreem Tanweer, Amna Batool, Zara Taimoor, Sundus Riaz, Syed Mohsin Ali

**Affiliations:** 1 Department of Biomedical Engineering and Sciences, School of Mechanical and Manufacturing Engineering, National University of Sciences & Technology, Islamabad 44000, Pakistan; n.ismara@hotmail.com (I.N.); tt1849@gmail.com (T.T.); amnabatoolx@gmail.com (A.B.); zarataimoor94@gmail.com (Z.T.); 13msbmessriaz@smme.edu.pk (S.R.); smohsin009@hotmail.com (S.M.A.); 2 Materials Engineering Department, School of Chemical & Materials Engineering, National University of Sciences & Technology, Islamabad 44000, Pakistan; nasir.ahmad@scme.nust.edu.pk

**Keywords:** cannabinoids, terpenes, polyethersulfone ultrafiltration membrane, antifouling performance, antibacterial activity

## Abstract

Plant phytochemicals have potential decontaminating properties, however, their role in the amelioration of hydrophobic water filtration membranes have not been elucidated yet. In this work, phytochemicals (i.e., cannabinoids (C) and terpenes (T) from *C. sativa*) were revealed for their antibacterial activity against different Gram-positive and Gram-negative bacteria. As such, a synergistic relationship was observed between the two against all strains. These phytochemicals individually and in combination were used to prepare polyethersulfone (PES) hybrid membranes. Membrane characterizations were carried out using scanning electron microscopy, Fourier transform infrared spectroscopy, energy-dispersive X-ray spectroscopy. Moreover, contact angle, water retention, surface roughness, mechanical testing, and X-ray florescence analysis were also carried out. According to results, the CT-PES hybrid membrane exhibited the lowest contact angle (40°), the highest water retention (70%), and smallest average pore size (0.04 µm). The hybrid membrane also exhibited improved water flux with no surface leaching. Quantitative bacterial decline analysis of the CT-PES hybrid membranes confirmed an effective antibacterial performance against Gram-positive and Gram-negative bacteria. The results of this study established cannabinoids and terpenes as an inexpensive solution for PES membrane surface modification. These hybrid membranes can be easily deployed at an industrial scale for water filtration purposes.

## 1. Introduction

Global health is confronted by the utilization of contaminated drinking water. Developing countries face huge challenges in cleaning water sources containing an apical fraction of pollutants from wastewater, sludge, sewage, and potentially toxic industrial effluents. In addition to this, bacterial clogging experienced by water pipelines leads to deplorable contamination consequences across the water supply system [1]. Consumption of this contaminated water is one of the key reasons for morbidity and mortality, especially in developing countries where multidrug resistance is already a great concern [2,3].

Extensive research efforts have been carried out to overcome the concern of drinking and wastewater contamination. In recent years, ultrafiltration (UF) membrane separation techniques have gained much attention as they are efficient, faster, and cost-effective than conventional separation techniques [4]. These membranes are being tailored and their surfaces modified to enhance the filtration capacity, increase hydrophilicity, and reduce fouling. Developments in this polymer membrane technology have suggested increased consumption of synthetic polymer membranes. Up until now, various types of UF membranes have been utilized for the elimination of bacteria, viruses, and toxic chemicals from contaminated water [5].

Despite having a range of merits over other conventional water treatment techniques and the capacity to execute several functions, membrane technology has some limitations in water treatment (i.e., biofouling, hydrophobicity, reduced water flux, etc.) [6]. Moreover, filtration of microscopic agents like bacteria and viruses may not be accomplished based on the composition and processing condition of the casting solution [7]. To modify and enhance characteristics of these membranes, the blending of different polymers, incorporation of organic compounds, and treatment of membranes with surfactants has been extensively applied [5].

Commercially, porous polymeric membranes (i.e., cellulose acetate, polyacrylonitrile (PAN), polyvinyl alcohol (PVA), polysulfone, polyvinylidene fluoride (PVDF) and polyethersulfone (PES)) are used for membrane fabrication [8]. Polyethersulfone (PES) is a FDA approved polymer that exhibits exceptional temperature, hydrolytic, oxidative, and chemical stability. Beholding mechanical and film forming properties, it is widely applied in microfiltration, ultrafiltration, and gas separation assemblies [9]. It is used for the separation of proteins, dialysis membranes, and infusion therapy membranes. Polluted water purification from the leather industry and other wastewater sources are reported to be effectively decontaminated by PES and its modified membranes [10].

Despite being effective in membrane preparations, the hydrophobicity of PES leads to the fouling of membranes as proteins, microbes, and other organic compounds tend to become attached to its hydrophobic surface. This results in a lower filtration efficiency and the poor filtration flux of PES membranes, which makes early cleaning or replacement of the membrane necessary [11]. Hydrophilic surface modification of the membrane serves as an effective solution to this problem. Several surface modification techniques have been developed to deal with the issue of biofouling such as coating, blending, grafting. etc. Among these modification techniques, blending is a facile method and is widely preferred [12]. Surface modification through the blending of PES with hydrophilic materials results in a hybrid polymer network in which comparatively smaller molecules are entrapped in the polymer mesh. This not only lowers membrane fouling, but also increases the water flux capacity of the membrane [13]. Most of the modifications made to endow antifouling characteristics in PES are of a superior nature and mostly applied for biomedical PES membranes. For large-scale purification, cost effective microfiltration and ultrafiltration membranes are required, which can be replaced after fouling.

Natural plant products have been used to impart antibiofouling characteristics to UF membranes due to their antibacterial activities and lower costs. Plant based additives such as curcumin [12], gum arabic [11], cyrene [8], etc., have been used as biofouling preventers of PES membranes. However, there is limited research in the use of phytochemicals for the fabrication of PES membranes.

In the last decade, *C. sativa* has been extensively cited for its medicinal importance. It is a fast growing plant with a rich repertoire of phytochemicals [14]. To the best of our knowledge, there is limited literature that is concerned with the fabrication of membranes with phytochemicals extracted from *C. sativa*. The main objective of the present study is to investigate the antibacterial and antibiofouling characteristics of cannabinoid and terpene blended PES membranes. For this purpose, cannabinoids and terpenes were first extracted from *C. sativa* and their synergistic antibacterial activity was analyzed. These phytochemicals were then blended with the PES membrane to prepare hybrid membranes. Hybrid and pure membranes were then characterized for their hydrophilicity and antibiofouling properties using Fourier transform infrared spectroscopy (FTIR), scanning electron microscopy (SEM), energy-dispersive x-ray spectroscopy (EDX), mechanical testing, contact angle, water retention, and surface roughness analysis. Moreover, water flux measurement and x-ray florescence (XRF) analysis for membrane leaching was also conducted. Finally, all membranes including hybrid and pure PES membranes were tested for their quantitative bacterial decline analysis against Gram-positive and Gram-negative bacteria. This study revealed cannabinoids and terpenes as an effective antibiofouling and antibacterial promotor of PES water filtration membranes. These membranes are a cost effective alternative to synthetically modified PES membranes and they can be installed at an industrial scale for drinking and wastewater purification.

## 2. Results and Discussion

### 2.1. Isolation of Cannabinoids and Terpenes from C. sativa 

First, cannabinoids and terpenes were extracted through the butanol and methanol extraction procedures, respectively. *C. Sativa* was selected in this work due to its extensive application in different fields of science (i.e., medicine, textile, pharmaceuticals, agriculture, etc.). In addition, extracts of *C. Sativa* have been described to possess antibacterial activity and their use in antibacterial finishing agents, functional textiles, and surgical devices has been reported previously. Phytochemicals present in this plant have been reported to interact synergistically to create an “entourage effect”, consequently improving the benefits of each phytochemical [14]. Among the wide variety of phytochemicals present in *C. sativa,* cannabinoids and terpenes were extracted in the present work. Terpenes belong to a group of hydrocarbons with multiple isoprene units, and are hydrophobic in nature, while cannabinoids belong to phenolic triterpenoids [15,16]. 

After extraction, the antibacterial activity of crude phytochemicals was realized. The results of the antibacterial activity of cannabinoids and terpenes are presented in Figure S1.

### 2.2. Synergistic Antibacterial Activity of Cannabinoids and Terpenes

**Figure 1:**
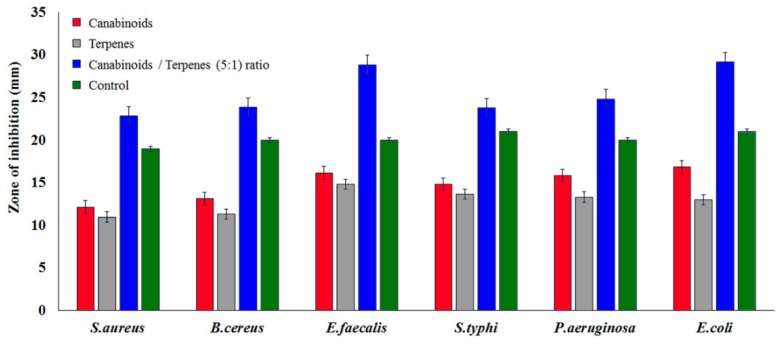
Synergistic antimicrobial activity of cannabinoids:terpenes (5:1 ratio).

For synergistic interaction, different combinational ratios (1:1 to 8:1) of cannabinoids:terpenes (C:T) and terpenes:cannabinoids (T:C) were tested at a concentration of 750 µg/disc. Cannabinoids and terpenes in a 5:1 ratio exhibited the highest zone of inhibitions against *E. coli* at 29 mm, followed by *E. faecalis* at 28.8 mm (Figure 1). This significant synergism was observed for all Gram-positive and Gram-negative bacteria.

### 2.3. Membrane Fabrication 

Using cannabinoids and terpenes, three different types of hybrid membranes were prepared: (i) a terpene blended PES membrane (T-PES); (ii) cannabinoid blended PES membrane (C-PES); and (iii) both cannabinoid and terpene (mixed in ratio 5:1) blended membrane (CT-PES). Surface and bulk modification of PES membranes were reported to enhance permeation and hydrophilicity of the membrane. Previously, various synthetic additives have been reported to improve PES membranes. Phenolic acid using the laccase enzyme has been reported to improve PES interaction with fouling agents. Acrylic acid and N-vinylpyrrolidone [13], poly (2-dimethylaminoethyl methacrylate [17], and aryl diazonium salts [18] have also endowed an antifouling property to the PES membranes. The blending of PES with silica nanoparticles and silver nanoparticles loaded membranes was also reported to supplement antibacterial property [19]. We selected crude cannabinoids and terpenes for surface modification, as they are ecofriendly, allow renewability, and offer low membrane fabrication costs when compared to other synthetic additives. 

### 2.4. Characterization

#### 2.4.1. Characterization of Cannabinoids and Terpenes 

To identify the functional groups and chemical bonds, FTIR analysis was carried out. The FTIR analysis of terpenes showed a characteristic C–H rock at 720 cm^−1^, which is characteristic for long chain alkanes. Peaks at 850–780 cm^−1^ show C–H bends, which confirmed the presence of alkenes, 1060–1040 cm^−1^ for O–H corresponds to primary alcohols, 1490–1380 cm^−1^ for C–C that can be aromatic, 1650–1600 cm^−1^ for C=C, 1725–1700 cm^−1^ for C=O, two peaks at 2980 and 2850 cm^−1^ for C–H, which belongs to the alkyl groups, and a wide band at 3500–3200 cm^−1^ for O–H appears to be for alcohol. All these peaks showed that *C. sativa* terpenes are complex phytochemicals with long hydrophobic chains. The results confirmed the cyclic structure with C=O and conjugate double bonds. This type of structure is common for terpenes from aroma producing plants. In *C. sativa,* most commonly, monoterpene myrcene, sesquiterpenes, α-humulene, and β-caryophyllene are present. Monoterpenes (i.e., α-pinene, linalool, limonene, sesquiterpenes bisabolol as well as (*E*)-β-farnesene) are also commonly present in *C. sativa* [20]. Characteristic peaks for C=C, C-H, and O-H also confirmed the isolation of terpenes from *C. sativa*.

Cannabinoids are phenolic triterpenoids that have hydroxyl groups and delocalized electrons and alkyl chains [20]. FTIR results of the extracted cannabinoids showed characteristic peaks at 720 cm^−1^ for hydrophobic alkyl chains, which were supported by two bands before 3000 cm^−1^. Additionally, there was C–H in plane bending at 1020 cm^−1^, 1480–1370 cm^−1^ for C–C, 1650–1600 cm^−1^ for C=C, two peaks at 2920 and 2850 cm^−1^ for C–H, and a wide band at 3500–3200 cm^−1^ for O–H. Characteristic peaks obtained through the FTIR analysis of the crude extract of cannabinoids identified characteristic chemical bonds present, and therefore confirmed their isolation. The most common cannabinoids present in the *C sativa* extracts were Δ9-tetrahydrocannabinolic acid (THCA) and cannabidiolic acid (CBDA) [20]. 

#### 2.4.2. Characterization of Pure PES and Hybrid Membranes 

##### Fourier Transform Infrared Spectroscopy (FTIR) analysis of Hybrid Membranes

**Figure 2:**
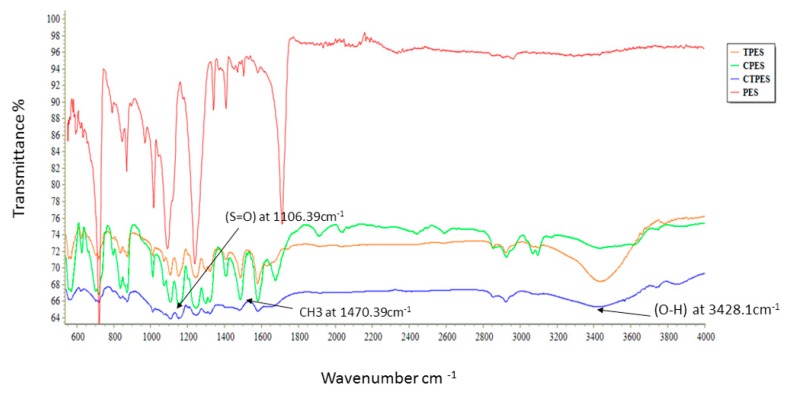
FTIR analysis of pure polyethersulfone (PES), cannabinoid and terpene embedded polyethersulfone (CT-PES), terpene embedded polyethersulfone (T-PES), and cannabinoid embedded polyethersulfone (C-PES) membranes.

Infrared spectra and characteristic bands observed in the pure and hybrid membranes lie in the range of 400–4000 cm^−1^. A comparison of the FTIR spectra of the hybrid membranes (i.e., C-PES, T-PES and CT-PES) with the pure PES membrane is provided in Figure 2.

For the pure PES membrane, the characteristic spectral bands were observed at 1150 cm^−1^ and 12,400 cm^−1^, which can be attributed to the (S=O) group and aromatic ether, respectively. Compared to the pure PES membrane, the hybrid membrane blended with crude extracts (CT-PES) comprised of new stretches at O–H at 3428 cm^−1^ and –CH3 at 1470 cm^−1^ stretches. In the hybrid membrane with terpenes, the wide OH stretch peak was sharper. The analysis of the FTIR spectrum of the pure PES and hybrid membranes suggested extensive resemblances, which ratified the blending of PES with cannabinoids and terpenes (Figure 2). As such, it is thought that blending and hydrophilization of the PES membrane occurs because of the coordination and hydrogen bond interaction between the sulfonic group and ether bond of the PES as well as to presence of hydroxyl groups in cannabinoids and terpenes [21]. 

##### Energy-Dispersive X-ray Spectroscopy Analysis (EDX)

Chemical composition and distribution of the chemical elements of interest in the pure PES and hybrid membranes were evaluated by EDX analysis. EDX analysis consisted of EDX spectra that provide peaks that correspond to all elements existing in the pure PES and hybrid membrane. Results of the EDX analysis are shown in Figure S2. Characteristic optical absorption peaks of unique energy were observed for all membrane types (i.e., PES, CT-PES, C-PES, T-PES). Characteristic carbon and sulfur picks were observed in all patterns, which confirms the blending of cannabinoids and terpenes in the PES membrane.

##### Morphological Analysis 

**Figure 3:**
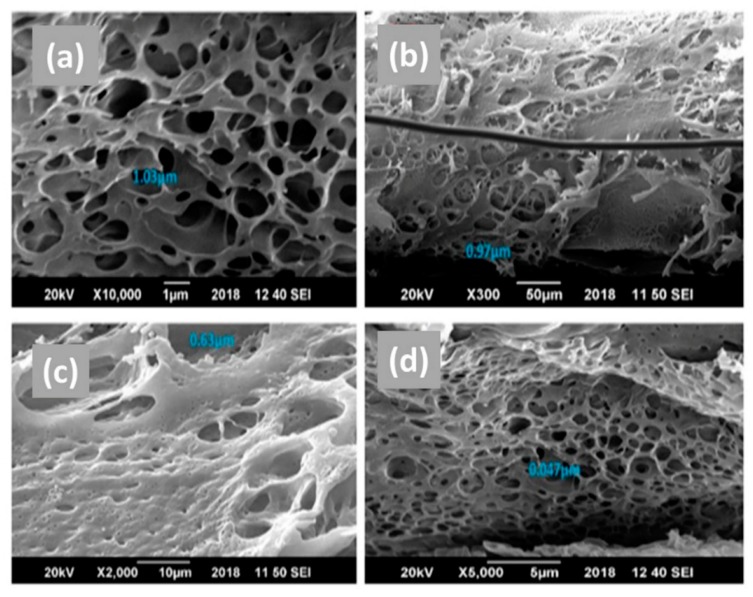
Scanning Electron Microscopy image showing morphology and pore size of the (**a**) pure polyethersulfone (PES), (**b**) cannabinoid embedded polyethersulfone (C-PES), (**c**) terpene embedded polyethersulfone (T-PES), and (**d**) cannabinoid and terpene embedded polyethersulfone (CT-PES), membranes.

**Figure 4:**
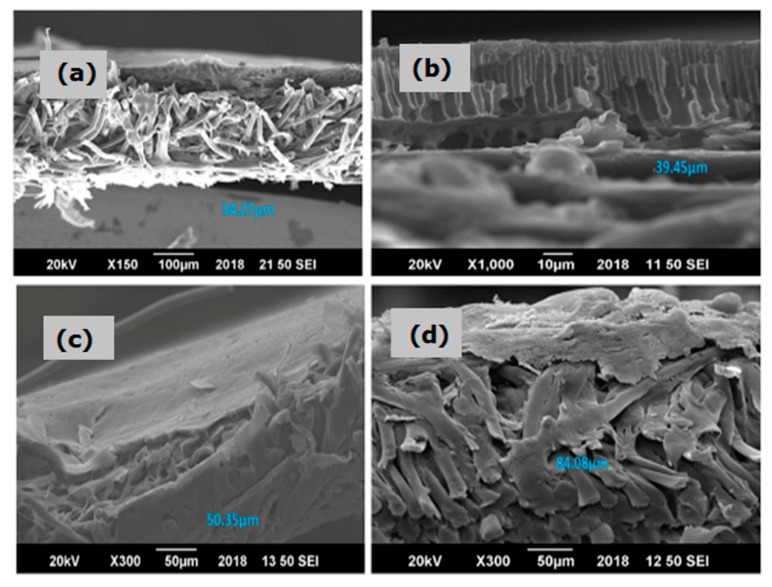
SEM image showing the morphology and width of the (**a**) pure polyethersulfone (PES) membrane, (**b**) cannabinoid embedded polyethersulfone (C-PES) membrane, (**c**) terpene embedded polyethersulfone (T-PES) membrane, and (**d**) cannabinoid and terpene embedded polyethersulfone (CT-PES) membrane.

To analyze the morphology of the pure PES and hybrid membranes, scanning electron microscopy (SEM) images were taken. The SEM images were then analyzed by ImageJ software ((NIH and LOCI, Madison, WI, USA)) to evaluate the average pore size and average thickness/width of the membranes. Cross-sectional images of pure PES along with hybrid membranes (i.e., C-PES, T-PES and CT-PES membranes) presenting the top view and side view are labelled in Figure 3 and Figure 4, respectively. Morphological analysis showed that all membranes exhibited a typical asymmetric structure. The top layer of the membranes was dense and the sublayer was porous. At the bottom of the membranes, fully developed macropores were also observed. However, repression in the formation of macropores was observed by the addition of cannabinoids and terpenes (Figure 4).

Membrane morphology revealed that the CT-PES membrane had the least average pore size among the pure PES and hybrid membranes (Table 1, Figure 3). The decrease in the average pore size of the CT-PES membrane may be attributed to an upsurge in the casting solution’s viscosity (i.e., PES reaching its threshold value). According to previous studies, at a definite threshold value, the casting solution becomes highly viscous, which results in a delay in demixing. This results in membranes with smaller pore sizes and less interconnecting channels [22]. Our purpose was to obtain smaller pore sizes in the membranes as irreversible pore fouling is least favorable in membranes with smaller pores, making them convenient for long-term water treatment operation. Conversely, membranes with a higher pore size are prone to blockage by organic materials and would require multiple chemical cleanings for long-term operations [23]. In addition, a smaller pore size of the membrane makes it selectively permeable, hence preventing smaller particles such as bacteria to move across the membrane.

Observation of the hybrid membrane’s surface morphology revealed the absence of any cracks on the surface and indicates that the surface of the hybrid membrane did not deteriorate due to the blending of cannabinoids and terpenes. However, there was a striking difference on the surface of the pure PES and hybrid membranes because to the alteration due to the incorporation of cannabinoids and terpenes. The blending of terpenes and cannabinoids with PES membranes with smoother surfaces are represented in Figure 3d.

The average width/thickness of the PES, C-PES, T-PES, and CT-PES membranes are provided in Table 1 and Figure 4. The maximum average thickness was observed for the CT-PES membranes. Improved thickness is attributed to the presence of hydrophilic additives (cannabinoids and terpenes) in the casting solution. It has been reported that increased concentration of hydrophilic additives may lead to an upsurge in the viscosity of the solution, which improves the thickness and compactness of the hybrid membranes. This happens due to the formation of complexes between the additive and polymer, which will reduce interaction between the polymer chains. Moreover, by increasing the concentration of hydrophilic additives, water penetration through the membrane may also increase. Increased thickness is also attributable to decreased porosity and the pore size of the hybrid membranes [24]. This trend was also observed in the C-PES, T-PES, and CT-PES hybrid membranes.

### 2.5. Mechanical Testing

To realize and compare the mechanical properties of the pure PES and hybrid membranes, the Young’s modulus was calculated. The Young′s modulus provided information about the stiffness of the hybrid membranes. Results of the Young’s modulus, maximum stress, and maximum strain are provided in Table 2 where the Young’s modulus, stress, and strain results showed the minimum value of Young’s modulus for the CT-PES membrane, followed by the C-PES, T-PES, and pure PES membranes (Figure S3). Similar results of a decrease in the Young’s modulus on the introduction of hydrophilic additives have also been observed in previous studies [25,26]. Reduction in tensile stress as well as Young′s modulus was related to the alteration in the structure of the membrane upon introducing phytochemicals. Reduction in tensile stress and Young’s modulus of the hybrid CT-PES membrane can be explained by the presence of fragile points in the membrane, which were created by macrovoids present in the skin top and porous sublayers of the hybrid membranes [27]. By tuning the concentration of PES and phytochemical additives, these macro voids can be avoided, which may assist in ameliorating the mechanical strength of the hybrid membranes [28].

### 2.6. Contact Angle, Water Retention, and Surface Roughness

The hydrophilicity and surface wettability of the hybrid membranes were evaluated by contact angle and water retention analysis of the hybrid membranes. Together, these parameters convey effective evidence about the hydrophilicity of the hybrid membranes upon surface modification [29]. The results of the contact angle and water retention analysis are provided in Table 3.

Contact angle measurement showed that the PES membrane had the maximum angle at 93° due to the hydrophobicity of the membrane, which was reduced to 60°, 56°, and 40° for the T-PES, C-PES and CT-PES membranes, respectively (Figure S4, Table 3). Decrease in the contact angle of the hybrid PES membranes after blending with cannabinoids and terpenes can be explained by the distribution of the hydrophilic ends of cannabinoids and terpenes on the surface of the membranes during the course of membrane formation [24]. During membrane formation, the hydrophobic ends of cannabinoids and terpenes interact with the hydrophobic methyl groups in the PES, which results in blending of the phytochemicals, while hydroxyl groups in the cannabinoids provide hydrophilic characteristics to the hybrid membranes [21]. Decrease in contact angle therefore confirms the hydrophilization of the hybrid membranes. Hydrophilization of the hybrid membranes resulted in improved water flux through the membrane with the counter entrance of organic contaminants through the membrane, therefore emancipating water from organic contaminants [30]. 

To measure the maximum moisture content that can be absorbed by a membrane, water retention analysis of the pure PES and hybrid membranes was carried out. The lowest water retention capacity (55%) was observed for the pure PES membrane among all membranes, which can be attributed to the hydrophobic character of the PES membrane [31]. The CT-PES membrane had the highest water retention capacities (70%) due to the presence of a polar phenol group in cannabinoids [15,16,21] that make the membranes relatively more hydrophilic, thus allowing water to pass through it. Water retention capacities in C-PES (58%) and T-PES (68%) were also observed to be higher when compared to pure PES.

The results for surface roughness are provided in Table 3. The surface of the CT-PES membrane was smooth in comparison to the pure PES membrane (Figure 3). Improved smoothness and reduced surface roughness in the hybrid membranes may result in improved antibiofouling characteristics. A higher surface roughness of the pure PES membrane compared to the modified membranes was also observed in previously undertaken studies [32,33]. It has been reported that in membranes with more surface roughness, fouling agents can get adsorbed in pores with a higher propensity compared to membranes with smoother surfaces [34]. Moreover, the increase of surface roughness may also cause irreversible fouling of PES membranes [35].

### 2.7. X-ray florescence Analysis for Surface Leaching 

The XRF analysis of the filtered water from the CT-PES membrane showed no possible leaching of cannabinoids and terpenes into filtered water. The issue of membrane leaching is considered to be a big limitation as it may lead to lower mechanical strength of the membrane along with reduced water flux through the membrane [36,37]. Leaching of the membranes could also cause threats for living organisms and consumers of the treated water [38]. No leaching of cannabinoids and terpenes from the hybrid CT-PES membranes confirms that these phytochemicals were strongly anchored to the PES matrix.

### 2.8. Membrane Flux

**Figure 5:**
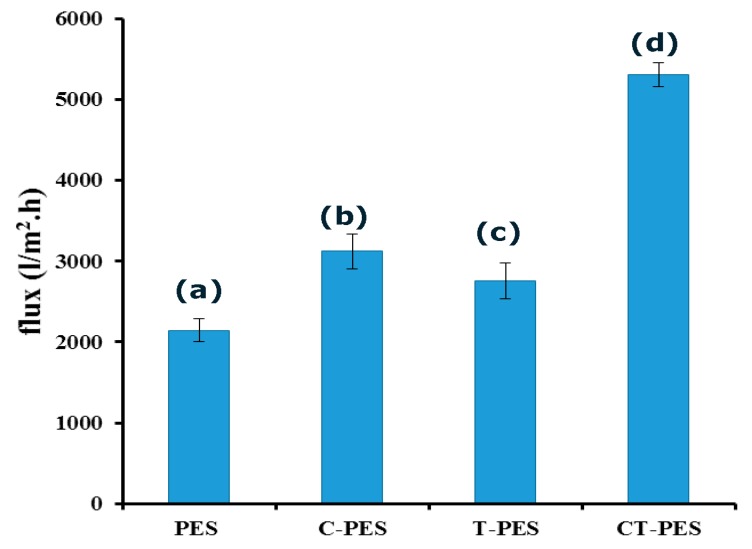
Graph showing membrane flux of the (a) pure polyethersulfone (PES) membrane, (b) cannabinoid embedded polyethersulfone (C-PES) membrane, (c) terpene embedded polyethersulfone (T-PES) membrane, and (d) cannabinoid and terpene embedded polyethersulfone (CT-PES) membrane.

Results for the membrane flux of the pure and hybrid membranes are provided in Figure 5. Water flux was observed to be the highest for the CT-PES membrane, followed by C-PES, T-PES, and pure PES. This increase in water flux was possibly ascribed to the hydrophilization of the hybrid membranes. Formation of enhanced hydrophilic characteristics augments the membrane surface’s affinity to water molecules despite the organic foulants, which may result in lower hydraulic resistance [38].

### 2.9. Quantitative Bacterial Decline Analysis of Membranes 

**Figure 6:**
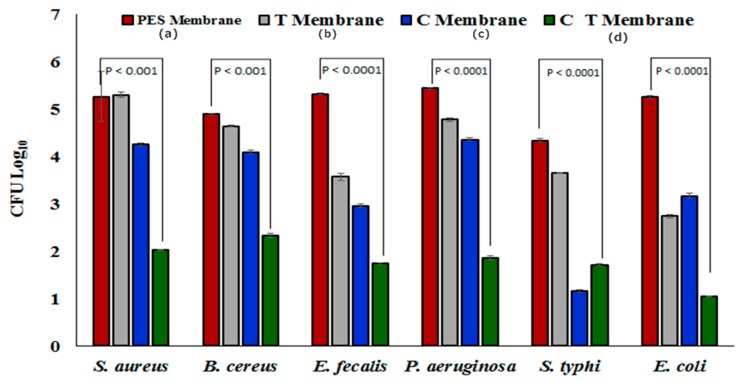
Graph showing bacterial decline in the (a) pure polyethersulfone (PES) membrane, (b) terpene embedded polyethersulfone (T-PES) membrane, (c) cannabinoid embedded polyethersulfone (C-PES) membrane, and (d) cannabinoid and terpene embedded polyethersulfone (CT-PES) membrane.

Results of the bacterial decline analysis are provided in Figure 6. Quantitative bacterial decline analysis of the membranes showed a decrease in the total colony forming units (CFU) of different bacterial strains before and after passing through the pure PES and hybrid membranes. For *S. aureus*, the pure PES membrane showed only a 30% decrease in the CFU, however, the hybrid membrane (CT-PES) exhibited an 88% decrease in CFU. This decrease was significantly higher than that of other hybrid membranes. For other bacteria such as *B. cereus* and *E. faecalis*, the CT-PES membrane showed an 88% and 80.5% decrease in the CFU compared to the PES membrane, which showed only a 2% and 7% decrease, respectively. Similarly for *S. typhi*, *P. aeruginosa*, and *E. coli*, the CT-PES membrane instigated decrease was 85%, 80%, and 85%, respectively, whereas for the PES membrane, the decrease was only 28%, 10%, and 50.8%, respectively. 

This considerable bacterial decline in the CT-PES hybrid membranes when compared to the pure PES membranes was attributable to the synergistic antibacterial activity of cannabinoids and terpenes (Figure 6), which has not only been reported in the present research, but also reported previously [39,40]. In addition to this, the antibacterial properties of the CT PES membranes could also be attributable to a rise in hydrophilicity, which may lead to lower the attachment of Gram-positive and Gram-negative bacteria on the membrane surface [41].

## 3. Materials and Methods

### 3.1. Materials

All chemicals were acquired from Sigma Aldrich (St Louis, MO, USA) unless indicated otherwise. Chemicals used for the extraction of phytochemicals and for the preparation of hybrid membranes included: polyethersulfone polymer (Ultrasone, Ludwigshafen, Germany), N-methyl-2-pyrrolidone (NMP), nutrient agar (MERK, München, Germany), and polyester support (MERK).

### 3.2. Plant Collection and Extraction of Phytochemicals

Collection of fresh leaves of *C. sativa* was carried out in the vicinity of the National University of Sciences and Technology, Islamabad, Pakistan. Collected leaves were washed thoroughly under tap water to remove impurities. Leaves were further washed under distilled water and were allowed to air-dry to remove moisture content. Dried leaves were then crushed using an electric grinder and sieved to obtain fine powder. Powder was kept under clean and dry polyethylene bags [42]. The extraction of cannabinoids and terpenes was then carried out, according to the following extraction procedure.

#### 3.2.1. Butanol Extraction of Cannabinoid

Plant powder weighing 15 g was decarboxylated at a temperature of 105 °C to activate the cannabinoids. These activated cannabinoids were extracted with butanol using a Soxhlet apparatus. The solution obtained after extraction was than filtered and refrigerated overnight for precipitation of waxes. After this, the supernatant was concentrated to half of its volume and extracted with 2% NaOH, thereby preventing any further oxidation. In the end, the aqueous layer was separated and the solvent was concentrated using a rotary evaporator [40].

#### 3.2.2. Methanol Extraction of Terpenes

Plant powder weighing 15 g was extracted with Soxhlet using methanol as a solvent. The obtained alcoholic extract was then concentrated to dryness using a rotary evaporator. The dried extract was than dissolved in 2N-HCl in a water bath, shaken, and then filtered. The acidic layer was adjusted to an alkaline pH [43].

### 3.3. Antibacterial Activity of Cannabinoids and Terpenes

Gram-positive bacterial strains used in this work included *Staphylococcus aureus* (ATCC 6538), *Bacilus cereus* (soil isolated), and *Enterococcus faecalis* (JH2-2, clinical isolate), while Gram-negative strains included *Salmonella typhi* (ATCC 6539), *Pseudomonas aeruginosa* (ATCC 9027), and *Escherichia coli* (ATCC 8739).

The antibacterial activity of different concentrations of cannabinoids and terpenes was tested by the disc diffusion method. For this purpose, first nutrient agar was transferred into Petri dishes and allowed to solidify. A 6 mm cork borer was used to create wells [44].

### 3.4. Fabrication of Mixed Matrix Polymer Membrane

Control and hybrid PES membranes were prepared by using the phase inversion method [12].

#### 3.4.1. Control PES Membrane Preparation

The control membrane (pure PES membrane) was made by mixing 18% (*w*/*w*) PES polymer (molecular weight: 232.258) in N-methyl-2-pyrrolidone (NMP) solvent. The mixture was stirred at 55 °C for 3 h, thereby allowing the polymer to dissolve completely, followed by continuous magnetic stirring for 24 h. The solution was cast upon a polyester support by a thin film applicator. The membrane was then allowed to immerse in the casting tray for about 10 to 15 min. Finally, the solidified membrane was air-dried.

#### 3.4.2. PES hybrid Membranes Preparation

The PES hybrid membranes were prepared by blending PES with crude extracts of cannabinoids and terpenes. Two different solutions were prepared for this purpose. Solution I contained 30 % (*w*/*w*) of the PES in NMP solvent. It remained on constant stirring at 55 °C for 3 h, ensuring that the polymer had completely dissolved. This was further followed by non-stop stirring for 24 h at 300 rpm. Solution II contained 4.2 % (*w*/*w*) of cannabinoid and terpenes (5:1 ratio) for the CT-PES membrane, crude cannabinoids for the C-PES membrane, and crude terpenes for the T-PES membrane in NMP solvent. Solution II was then stirred for 60 min upon a magnetic stirrer followed by sonication for 15 min. The sonication step was repeated thrice. Both Solutions II and I were than mixed together and cast. After immersing in water, the membranes were air-dried for 48 h before further analysis.

### 3.5. Characterization of Crude Cannabinoids and Terpenes

To characterize the presence of the characteristic functional groups and bonds in crude extracts of cannabinoids and terpenes, FTIR analysis was conducted. To carry out this analysis, crude extracts of cannabinoids and terpenes were smeared on KBr and compressed into a thin pellet. Infrared spectra was than recorded with a FTIR spectrometer (Perkin Elmer spectrum 100 instrument (Waltham, MA, USA) between 4000–400 cm^−1^.

### 3.6. Characterization of Hybrid Membranes

#### 3.6.1. FTIR Analysis

To characterize the occurrence of cannabinoids and terpenes in hybrid membranes, FTIR spectra of the pure PES membrane and hybrid membranes (i.e., CT-PES, T-PES, and C-PES) were measured. To carry out our FTIR of pure and hybrid membranes, small pieces from each membrane were dried for more than 48 h at room temperature and then compressed into a thin KBr pellet. Infrared spectra was than recorded between 4000–400 cm^−1^.

#### 3.6.2. EDX Analysis

EDX (HITACHI, Tokyo, Japan) analysis was carried out to investigate the elemental composition and functional groups in the pure and hybrid PES membranes, thereby confirming the blending of phytochemicals to PES. This analysis consisted of EDX spectra indicating the peaks corresponding to all elements present in the membrane.

#### 3.6.3. SEM

The influence of surface modification on the morphology of the hybrid membrane was studied using SEM (HITACHI, Tokyo, Japan). To observe the surface morphology, small pieces (0.25 cm^2^) of the test membranes were cut and conditioned in liquid nitrogen. Under vacuum, these test membranes were further coated with gold by sputter coating. After coating, the sliced margin of membrane was observed perpendicular to the cut plane by SEM. Surface and cross sectional micrographs were obtained. To obtain average pore size and membrane thickness, ImageJ software (NIH and LOCI, Madison, WI, USA) was utilized.

### 3.7. Mechanical Testing

Mechanical testing of pure PES and hybrid membranes was carried out using a SHIMADZU AG-Xplus series (Kyoto, Japan) Universal Testing Machine (UTM). To find out the mechanical properties, the uniaxial tensile testing method was used [45]. In the loading frame of the testing machine, the opposite ends of the membrane were gripped. At the gauge length of 100 mm, the membrane was stretched at a constant speed of 50 mm/min. The same procedure was applied to all membranes and on average, five readings were obtained for each membrane (i.e., pure PES, T-PES, C-PES, and CT-PES membrane). A software generated stress–strain graph was obtained and the Young’s modulus (€), maximum stress (B), and maximum strain (ε) of the membranes were recorded for each membrane.

### 3.8. Contact Angle Water Retention and Surface Roughness

Hydrophilicity and surface wettability of the pure and hybrid membranes were studied by contact angle analysis using the sessile drop method. At room temperature, approximately 10 μL of deionized water (DI) was suctioned by micro syringe and placed on the surfaces of the pure and hybrid membranes. Images were than taken using a camera. To determine contact angle, curve-fitting analysis was carried out on captured images using CAM 2008 software (Delcam, Birmingham, England, UK) [46]. Static contact angles were measured at four different positions on each membrane surface and by averaging these contact angles, the final water contact angle was obtained for pure and hybrid PES membranes.

To investigate the water retaining capacity of the pure and hybrid membranes, water retention analysis was carried out. For water retention analysis, 1 g of pure PES and hybrid membranes were soaked in distilled water for 24 h. The wet weight of each membrane was recorded. Membranes were then oven dried for 12 h. Dry weight of each membrane was recorded afterward [46]. Based on these measurements, the percentage water content of each membrane was calculated by using the following equation and five readings were recorded to ensure the accuracy of results.

(1) $$\%\;age\;of\;water\;retained\;content\;=\frac{\mathrm{Wet}\;\mathrm{Weight}\;-\;\mathrm{Dry}\;\mathrm{weight}\;}{\mathrm{Wet}\;\mathrm{weight}}\;\times \;100$$

Surface roughness of the pure PES and hybrid membranes was measured using the non-contact optical profilometer NONOVEA PS50 (Nanovea, Irvine, CA, USA). By using the stitching algorithm, a 0.187 × 0.140 mm area was scanned. Data from the surface image obtained for each membrane were processed by the optical profiler’s software (Nanovea, Irvine, CA, USA). Five profiles were recorded for each membrane and arithmetical mean surface roughness values (Ra) were estimated from an average of five profiles. Surface roughness was measured in µm [47].

### 3.9. X-ray Fluorescence (XRF) Test

The XRF test was carried out using a Joel JSK-3202M Element analyzer (Joel, Tokyo, Japan) to realize the leaching of cannabinoids and terpenes from hybrid membranes to the filtered water.

### 3.10. Membrane Flux Test

To find out the membrane flux, the membrane flux test was carried out. The filtrate assembly was arranged at room temperature with constant pressure of 60 cm-hg for 120 min. A sample of 0.025 m^2^ of membrane was tested under this assembly [46]. Membrane flux values provide an idea about the efforts required to produce the permeate and to compare the initial performance of the pure and hybrid membranes. The following equation was used to calculate the water flux of the pure PES and hybrid membrane:

(2) $$J=\frac{\Delta v}{A\Delta T}$$

where *J* is the pure water flux (L/m^2^ h); Δ*V* is the cumulative volume of permeate (L); A is the surface area of membrane (m^2^); and ΔT is the duration of filtration (h).

### 3.11. Quantitative Bacterial Decline Analysis

Quantitative bacterial decline analysis was carried out to look for the bactericidal activity of the pure and hybrid membranes. This was performed by filtering water through each membrane using the dead end filtration assembly. The main purpose of this analysis was to look for the effectiveness of hybrid membranes to block and remove bacteria in the water. Bacterial cultures of Gram-positive and Gram-negative bacteria were grown for six hours and diluted to 10^−6^ with sterile DI water. This feed was passed through the hybrid membranes at a pressure of 150 mmHg. After that, the filtrate water obtained was spread over nutrient agar and further tested for disinfection. Agar plates were than incubated at the temperature of 37 °C for 24 h duration. Original bacterial dilutions 10^−6^, were taken as a positive control. After the incubation step, the total colony forming units CFU mL^−1^ of the filtered water were compared with the control.

### 3.12. Statistical Analysis

IBM SPSS statistics software V. 25 (IBM corporation, New York, NY, USA) was used for the analysis.

## 4. Conclusions

In this research, cannabinoids and terpenes from *C. sativa* were used for the first time in the surface modification of PES membranes to improve its antibacterial and antibiofouling properties. The antibacterial assessment showed synergistic antibacterial activity of these phytochemicals in crude form. The hybrid CT-PES membrane was obtained after blending cannabinoids and terpenes with PES; this membrane possessed improved antibacterial and antibiofouling properties. In the CT-PES membrane, a 5% decrease in the average pore size was observed when compared to the pore size of the pure PES membrane. The contact angle of the hybrid membrane was also reduced to approximately 53% when compared to the pure PES membrane, which confirmed improved hydrophilicity. The hybrid membrane comprised a smooth surface and showed better water retention and improved water flux. Improvement in the properties of the PES membrane after blending can be ascribed to the chemical composition of cannabinoids and terpenes, which contain hydroxyl groups that arrange themselves to the surface, consequently increasing the hydrophilicity of the hybrid membrane. Quantitative bacterial decline assessment showed limited growth of Gram-positive and Gram-negative bacteria on the CT-PES membrane surface, which means that the hybrid membrane also possessed antibacterial properties. This study concluded that cannabinoids and terpenes are promising phytochemicals for PES membrane modification. Blending these phytochemicals will enhance the performance of ultrafiltration membranes for water treatment applications. These hybrid membranes are inexpensive when compared to other synthetic membranes and can be easily deployed at an industrial level for water filtration and purification purposes.

## Figures and Tables

**Table 1:** Average width along with the average pore size of pure polyethersulfone (PES), terpene embedded polyethersulfone (T-PES), and cannabinoid embedded polyethersulfone (C-PES), cannabinoid and terpene embedded polyethersulfone (CT-PES) membranes.

Sr. No.	Membrane Type	Average Width (µm)	Average Pore Size (µm)
**1**	PES	34.27	1.03
**2**	T	39.45	0.97
**3**	C	50.35	0.63
**4**	CT	84.08	0.047

**Table 2:** Young’s modulus, stress, and strain results for the PES, T-PES, C-PES, and CT-PES membranes.

Sr. No.	Membrane Type	Average Width (µm)	Average Pore Size (µm)
**1**	PES	34.27	1.03
**2**	T	39.45	0.97
**3**	C	50.35	0.63
**4**	CT	84.08	0.047

**Table 3:** Average contact angle and water retention and surface roughness of pure PES and hybrid membranes.

Sr. No.	Membrane	Average Contact Angle	Water Retention (%)	Surface Roughness Ra (nm)
**1**	PES	93	55	36,880
**2**	C-PES	60	58	7650
**3**	T-PES	56	68	4332
**4**	CT-PES	40	70	2170

## Supplementary Materials

The following are available online at https://www.mdpi.com/1420-3049/25/3/691/s1, Figure S1(a,b): Antibacterial activity of Cannabinoids against Gram negative and Gram positive bacteria., Figure S1(b, c): Antibacterial activity of Terpenes against Gram negative and Gram positive bacteria, Figure S2: EDX analysis of (a) pure Polyethersulphone (PES) membrane, (b) Cannabinoid and Terpene embedded polyethersulphone (C+T-PES) membrane (c) Terpene embedded polyethersulphone (T-PES) membrane and (d) Cannabinoid embedded polyethersulphone (C-PES) membrane, Figure S3: Stress vs strain graph for (a)Pure polyethersulphone (PES) membrane, (b) Terpene embedded polyethersulphone (T-PES) membranes, (c) Cannabinoid embedded polyethersulphone (C-PES) membranes and (d) Cannabinoid and Terpene embedded polyethersulphone (C+T-PES) membranes, Figure S4: Contact angle analysis of (a)Pure polyethersulphone (PES) membrane, (b) Cannabinoid embedded polyethersulphone (C-PES) membranes,(c) Terpene embedded polyethersulphone (T-PES) membranes and (d) Cannabinoid and Terpene embedded polyethersulphone (C+T-PES) membranes.
