# Supplementary material for: Cannabinoids and Terpenes as an Antibacterial and Antibiofouling Promotor for PES Water Filtration Membranes
Source: Molecules. 2020 Feb 6;25(3):691. doi: 10.3390/molecules25030691 (PMC7037186; doi:10.3390/molecules25030691)
Supplement: Supplementary file 1 [file molecules-25-00691-s001.pdf]

# Supplementary Materials

# Cannabinoids and Terpenes as an Antibacterial and Antibiofouling Promotor for PES Water Filtration Membranes

**Ismara Nadir [1], Nosheen Fatima Rana [1,*], Nasir Mahmood Ahmad [2] and Tahreem Tanweer [1], Amna Batool [1], Zara Taimoor [1], Sundus Riaz [1], Syed Mohsin Ali [1]**

[1] Department of Biomedical Engineering and Sciences, School of Mechanical and Manufacturing Engineering, National University of Sciences & Technology, Islamabad, Pakistan; n.ismara@hotmail.com (I.N.); tt1849@gmail.com (T.T.); amnabatoolx@gmail.com (A.B.); zarataimoor94@gmail.com(Z.T.); 13msbmessriaz@smme.edu.pk (S.R.); smohsin009@hotmail.com (S.M.A.)

[2] School of Chemical & Materials Engineering, National University of Sciences & Technology, Islamabad, Pakistan; nasir.ahmad@scme.nust.edu.pk (N.M.A.);

* Correspondence: nosheen.fatima@gmail.com; Tel.: +925190856074; Fax: +92-90856001

### Antibacterial Activity of Cannabinoids and Terpenes

The extracted phytochemicals were investigated for their antibacterial activity. It has been reported that the number of colony-forming units of *P. aeruginosa*, *E. faecalis*, and *E. coli* decreased on treatment with sodium hypochlorite. However, increasing the concentration and exposure time did not eliminate these bacteria. Therefore, they were specially included in the antibacterial test. The antimicrobial activities of crude cannabinoids and terpenes against Gram-positive and Gram-negative bacteria are presented in Figures S1a–d. The results obtained for the crude cannabinoid indicated that *E. coli* and *E. feacalis* showed the maximum inhibition zone values of 17 mm and 16.5 mm. The crude terpenes extract expressed a maximum inhibition zone against *S. typhi* and *E. faecalis* at 16 mm and 15.8 mm, respectively. These zones of inhibitions were obtained at higher concentrations of 50 mg crude compound $mL^{-1}$ corresponding to 500 μg/disc. Chloramphenicol formed an effective zone of inhibition in the range of 19 to 20 mm for Gram-positive bacteria and 20 to 21 mm against Gram-negative bacteria.

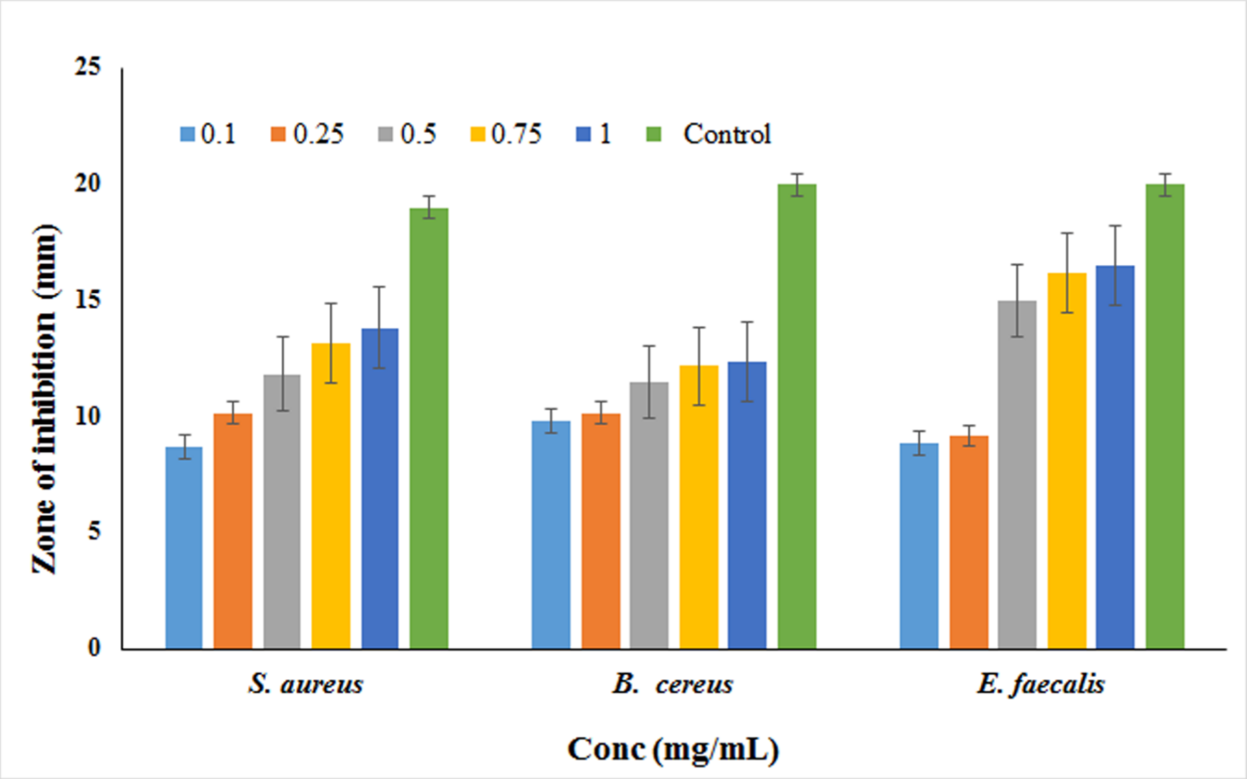


**Figure S1.** (**a**) Antibacterial activity of cannabinoids against Gram-negative bacteria.

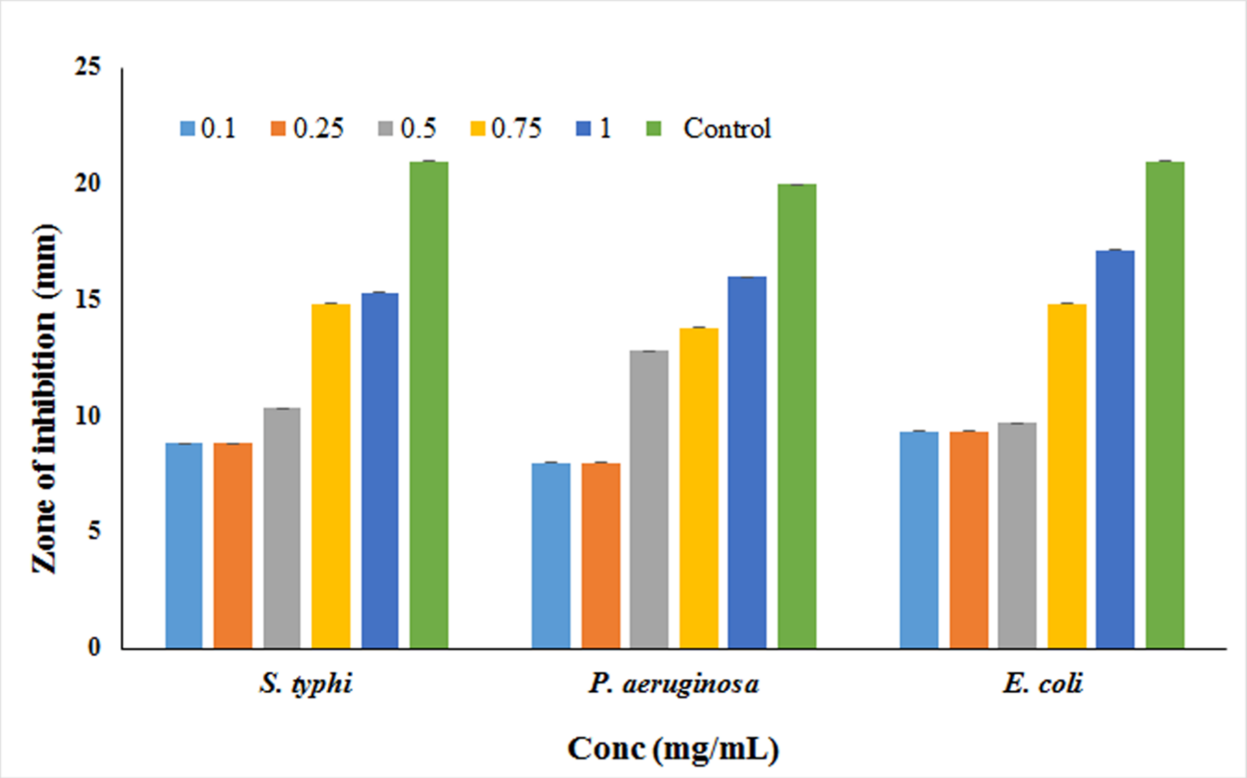


**Figure S1.** (**b**) Antibacterial activity of cannabinoids against Gram-positive bacteria.

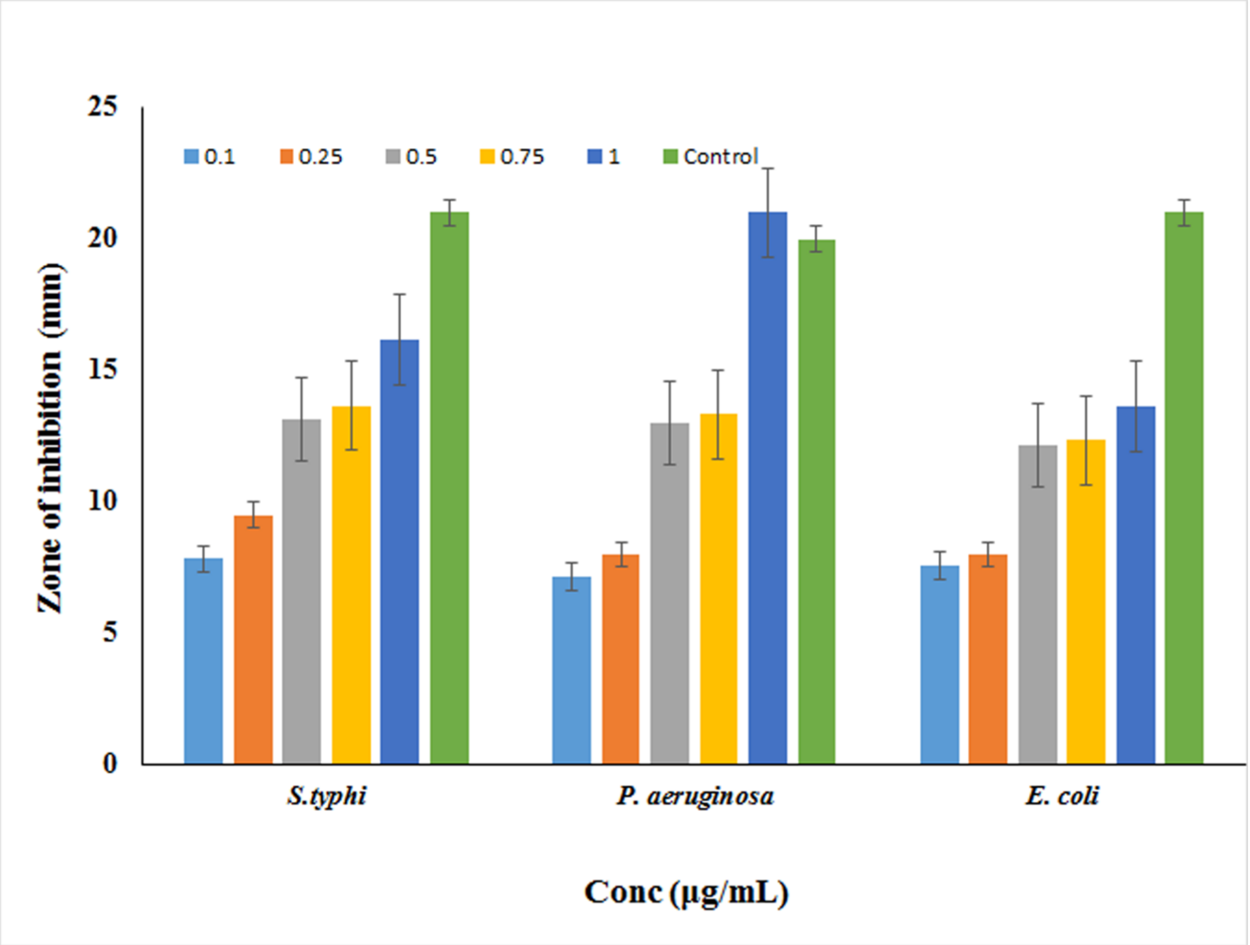


**Figure S1.** (**c**) Activity of terpenes against Gram-negative bacteria.

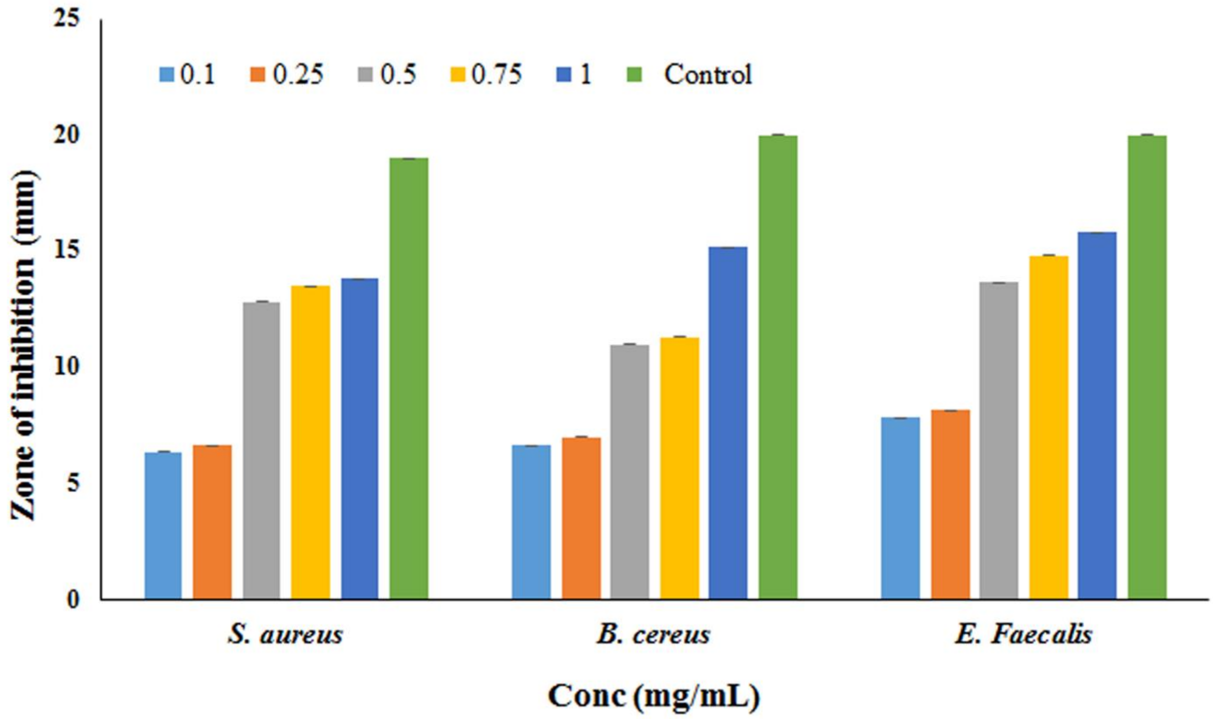


**Figure S1.** (**d**) Antibacterial activity of terpenes against Gram-positive bacteria.

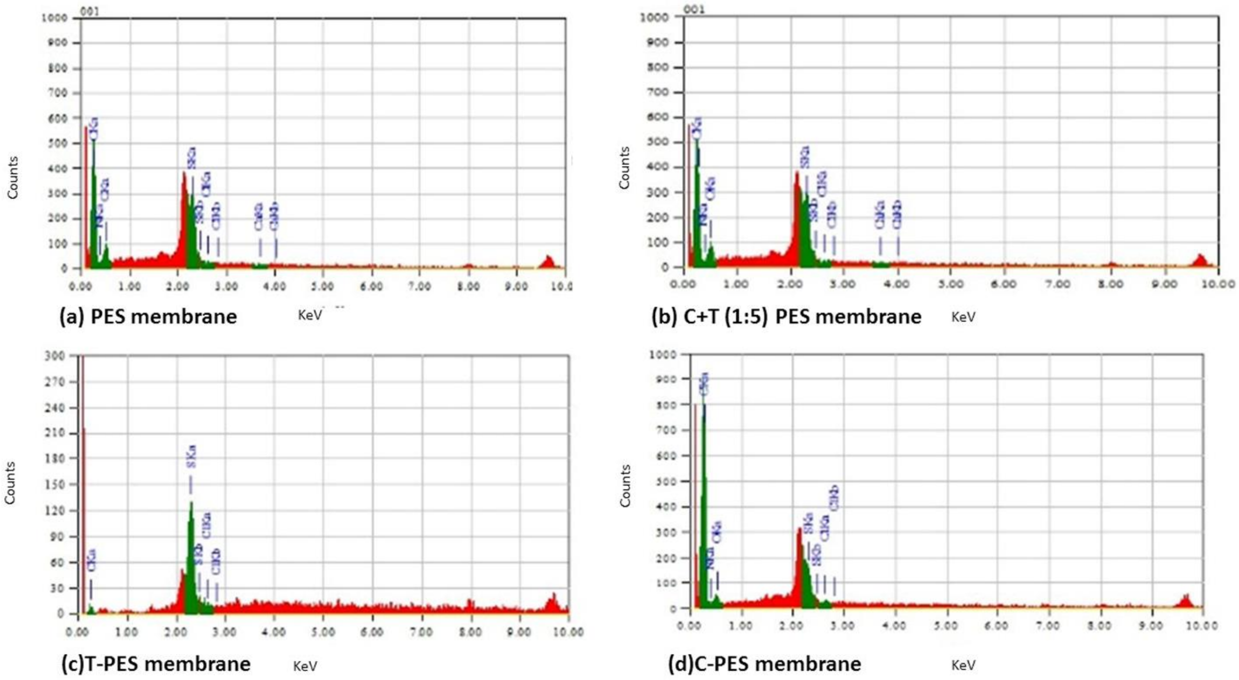


**Figure 2.** EDX analysis of (**a**) pure polyethersulfone (PES) membrane, (**b**) cannabinoid and terpene embedded polyethersulfone (C+T-PES) membrane, (**c**) Terpene embedded polyethersulfone (T-PES) membrane, and (**d**) cannabinoid embedded polyethersulfone (C-PES) membrane.

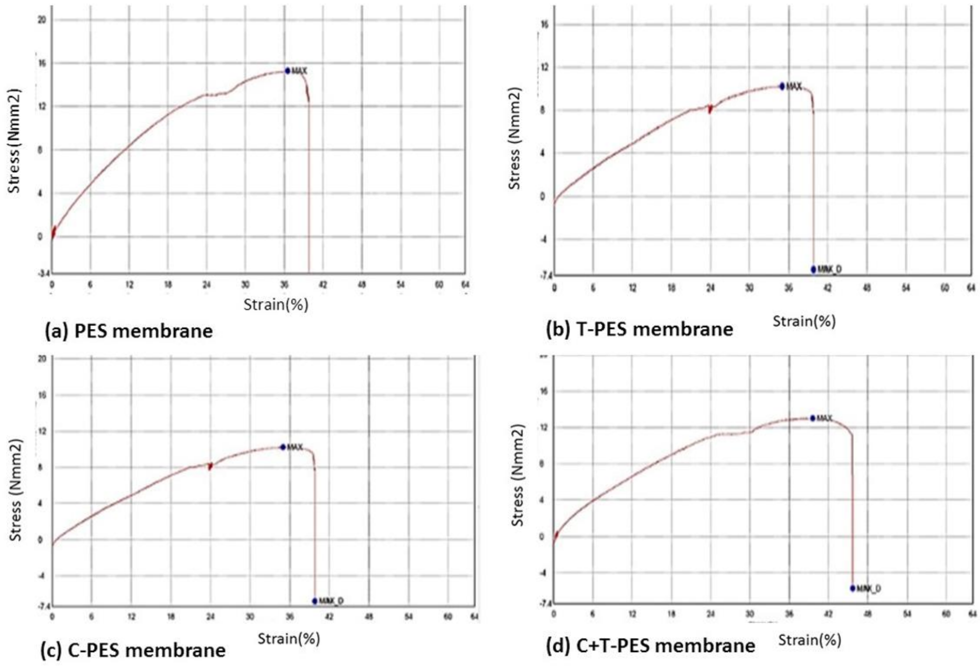


**Figure 3.** Stress vs. strain graph for (**a**) pure polyethersulfone (PES) membrane, (**b**) terpene embedded polyethersulfone (T-PES) membranes, (**c**) cannabinoid embedded polyethersulfone (C-PES) membranes, and (**d**) cannabinoid and terpene embedded polyethersulfone (C+T-PES) membranes.

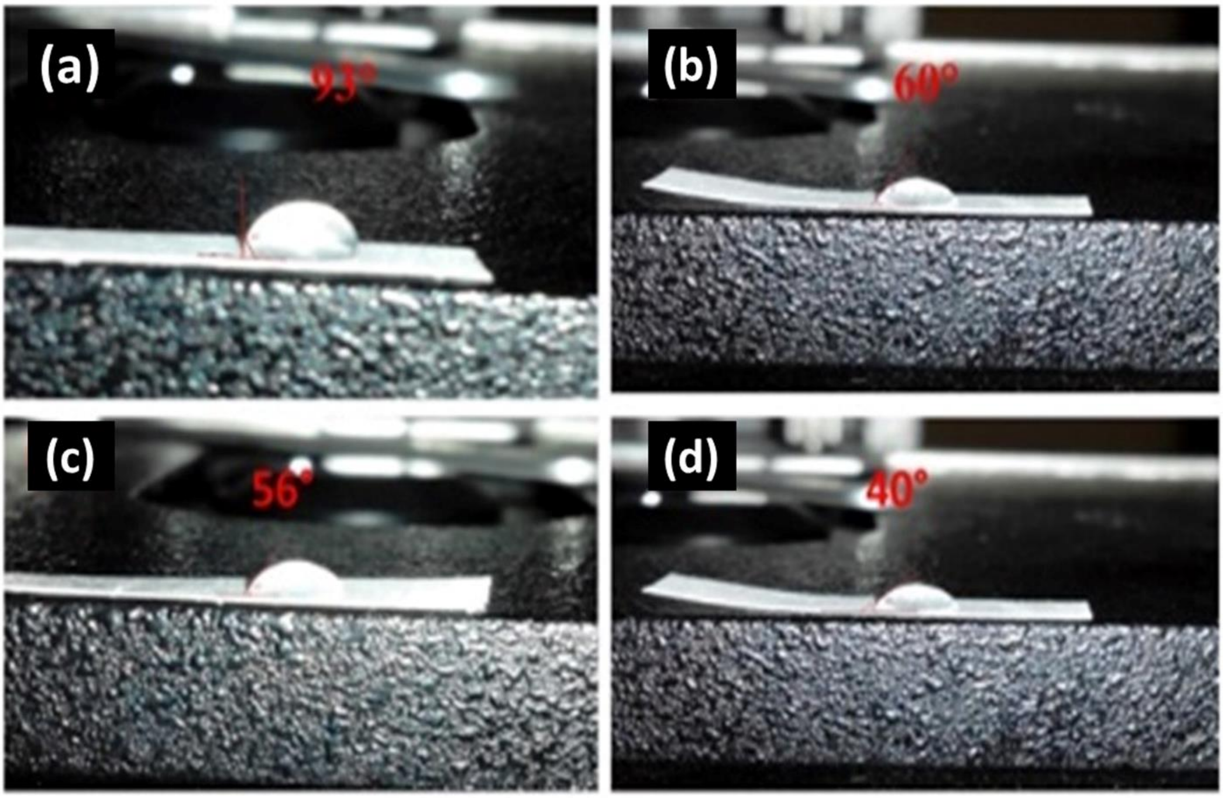


**Figure 4.** Contact angle analysis of (**a**) pure polyethersulfone (PES) membrane, (**b**) cannabinoid embedded polyethersulfone (C-PES) membranes, (**c**) terpene embedded polyethersulfone (T-PES) membranes, and (**d**) cannabinoid and terpene embedded polyethersulfone (C+T-PES) membranes.
